# The Four Horsemen of the Apocalypse: Tropical Medicine in the Fight against Plague, Death, Famine, and War† †Presidential address given at the 60th Annual Meeting of the American Society of Tropical Medicine and Hygiene, December 7, 2011, Philadelphia, Pennsylvania.

**DOI:** 10.4269/ajtmh.2012.11-0814

**Published:** 2012-07-01

**Authors:** Peter J. Hotez

**Affiliations:** National School of Tropical Medicine, Sabin Vaccine Institute, and Texas Children's Hospital Center for Vaccine Development, Departments of Pediatrics and Molecular Virology and Microbiology, Baylor College of Medicine, Houston, Texas

## Introduction

I want to thank Dr. Lance Gordon for his very generous introduction. I was especially thrilled that Lance was able to make introductory remarks this evening because he bridges two of the most important institutions in my professional life. Lance previously served on the Board of the Sabin Vaccine Institute, an extraordinary global health institution that I have been associated with for the last 12 years, and he recently began a new position with the Bill & Melinda Gates Foundation. Not only has the Gates Foundation generously supported our work on the human hookworm vaccine for the past decade, but in addition Gates, together with Sabin, has introduced me to some of my most important scientific mentors, including Drs. Philip K. Russell, Ciro de Quadros, Regina Rabinovich, and Jan Agosti. It is also through the Sabin Vaccine Institute that I met some other extraordinary individuals including H. R. Shepherd, the founding Sabin Board Chair who died this year at the age of 89; Ambassador Michael Marine, (Sabin CEO); Brian Davis (Sabin COO); Mrs. Heloisa Sabin; and the current Sabin Board Chair, Mort Hyman, who is a unique combination of mentor and friend.

In 2011, we relocated the laboratories of the Sabin Vaccine Institute to Texas Children's Hospital (TCH) in Houston to become the first product development partnership (PDP) embedded in an academic health center.^1^ This association came about through the visions of Dr. Mark Kline, the TCH Physician-in-Chief (an impressive global health advocate who created BIPAI, the Baylor International Pediatric AIDS Initiative), and Mark Wallace the TCH Chief Executive Officer. Simultaneously under the leadership of Dr. Paul Klotman, Baylor College of Medicine (BCM) President and CEO (also an ardent advocate and champion of global health), we have established a unique National School of Tropical Medicine to train a new generation of healthcare professionals in this area.^2^ Our tropical medicine clinic at the Ben Taub General Hospital (also linked with BCM) has opened and already we are seeing patients with Chagas disease, cysticercosis, and elephantiasis. We believe that we have uncovered a hidden burden of neglected tropical diseases in Texas!^3^

This year's annual meeting in Philadelphia has been extraordinary for a number of reasons. This afternoon, I learned from Karen Goraleski (American Society of Tropical Medicine and Hygiene [ASTMH] Executive Director) that we have broken all meeting attendance records to date. I want to use this opportunity to thank Karen for her extraordinary leadership in her first year with ASTMH, as well as the hard work of Judy DeAcetis and the rest of the Sherwood staff. The coming year will be an exciting one with Dr. James Kazura as your new President.

I hope to use this evening's ASTMH Presidential Address to highlight three important elements of tropical medicine that our Society must consider as we enter the second decade of this new century. First, I will address important aspects of tropical diseases that go beyond their health impact and examine tropical infections in a larger context of their economic and geopolitical effects. Second, given this level of global importance I want to call on the tropical disease community to consider a broad and “audacious” goal to eliminate the tropical infections affecting the world's poor. Finally, I hope to highlight a new opportunity we have to address tropical disease elimination goals as a means to implement international science diplomacy.

## The Four Horsemen Of The Apocalypse: Pestilence, Death, Famine, And War

**Figure 1.**
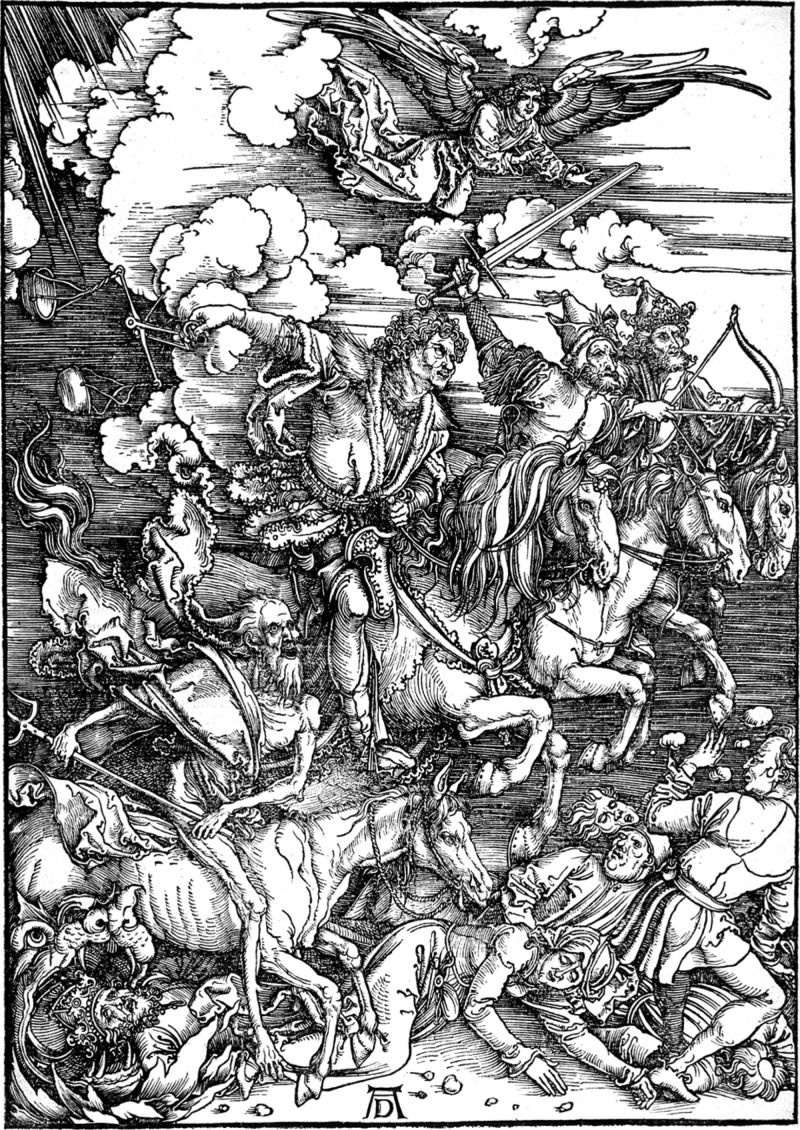
Woodcut of the Four Horsemen of the Apocalypse, ca. 1497–1498, Albrecht Dürer (German, 1471–1528).

To highlight the devastation wrought by the tropical diseases I invoke an apocalyptic vision found in the last book of the New Testament. The Four Horsemen of the Apocalypse ride on black, white, red, and pale horses, with each symbolizing a distinct aspect of the Last Judgment. One of the better known representations of the Four Horsemen is from a late 15th century woodcut by the gifted and psychological artist, Albrecht Dürer (Figure 1

). There are differing accounts and interpretations of the meaning of each mounted horse, but for this evening we will focus on their representations as pestilence, death, famine, and war.

### Pestilence.

The tropical diseases are the most common infections of the world's poorest persons, a group sometimes known as the “bottom billion,” referring to the estimated 1.4 billion persons that live below the World Bank poverty figure of US$1.25 per day.^1^,^4^ A new list of the world's major tropical infections ranked according to estimates of their prevalence in the biomedical literature is shown in (Table 1 ).^4^–^16^ Heading the list are the three major soil-transmitted helminth infections and schistosomiasis, with more than 500 million cases of each disease; followed by more than 100 million cases of amebiasis, malaria, lymphatic filariasis, and dengue; tens of millions of cases of trachoma, strongyloidiasis, onchocerciasis, food-borne trematode infections, and typhoid fever; and up to 10 million cases or more of leishmaniasis and Chagas disease.^4^–^16^ Many of these tropical infections, especially helminth infections and trachoma, are chronic conditions affecting persons for years or even their entire lives. There are approximately 3–4 billion cases of tropical infections worldwide, but these diseases are disproportionately shared between the bottom billion. By this statement I mean that much of the bottom billion is polyparasitized and therefore infected simultaneously with several tropical diseases, such as hookworm with malaria, schistosomiasis, or strongyloidiasis; onchocerciasis with loiasis; and ascaraisis with trichuriasis.^17^ Moreover, certain tropical infections such as female genital schistosomiasis or malaria appear to increase susceptibility to human immunodeficiency virus/acquired immunodeficiency syndrome (HIV/AIDS).^17^

Most of the diseases on this list, with the exception of *Plasmodium falciparum* malaria, are also known as neglected tropical diseases (NTDs).^4^,^9^,^13^,^17^–^19^ The conceptualization of these conditions as NTDs was put into the peer-reviewed biomedical literature in 2005 and 2006,^17^–^19^ and refers to them as a group of chronic parasitic and related tropical infections that are highly disabling, disfiguring, and stigmatizing.^14^ Many of the NTDs are non-emerging infections and have afflicted humankind for centuries, and their descriptions are found in ancient texts.^19^ In the 15th century, Dürer might have recognized several NTDs such as ascariasis and leprosy. The World Health Organization (WHO) has recently created a list of 17 common NTDs,^9^ and the journal *PLoS Neglected Tropical Diseases* recognizes an expanded list of almost 40 NTDs. A common element of the NTDs, and one that we will return to later, is their ability to actually cause poverty because of their profound effects on child growth and cognitive and intellectual development, pregnancy outcome, and agricultural worker productivity.^10^,^13^ The NTDs are also the most common infections of girls and women living in poverty.^20^ Together, the NTDs represent the most prevalent adverse health conditions among the world's poor living in low- and middle-income countries (LMICs). I often tell my lay colleagues, the NTDs are the “most important diseases you never heard of.”

### Death.

The tropical diseases are also important global causes of death. A new list of the world's tropical infections ranked according to the number of persons they kill annually is shown in (Table 2 ).^5^–^8^,^15^–^17^,^21^–^23^ To no one's surprise, malaria heads the list with more than 600,000 people dying annually, mostly children from *P. falciparum* infection, followed by more than 100,000 deaths annually from schistosomiasis, typhoid fever, and cholera, and tens of thousands of deaths from kinetoplastid infections (leishmaniasis and Chagas disease), amebiasis, dengue, and soil-transmitted helminthiases.^5^–^8^,^15^–^17^,^21^–^23^ In all, an estimated 1.5 million persons die each year from tropical infections. To put this number in perspective, together the tropical infections kill more persons than the number of children who die annually from lower respiratory tract infections,^23^ and together the tropical infections cause almost as many deaths as the 1.8 million people who died from HIV/AIDS in 2010.^24^

### Famine.

As I indicated earlier, the impact of tropical diseases goes beyond their adverse effects on human health. In my book *Forgotten People, Forgotten Diseases: The Neglected Tropical Diseases and their Impact on Global Health and Development*, I credit Professor Jeffrey Sachs and his *Report of the Commission on Macroeconomics and Health* for first highlighting the economic importance of malaria and other diseases.^25^ In my book and in subsequent papers, I summarize the stealth ability of NTDs to impair child development and agricultural productivity.^1^,^13^,^25^ Many of the adverse health effects of the NTDs operate through their ability to impair human nutrition. Some of the effects of NTDs on promoting hunger and even famine is summarized in Table 3 . Diseases such as lymphatic filariasis have been shown to reduce agricultural productivity in LMICs such as in Ghana and India; blinding eye disease from trachoma and onchocerciasis disables agricultural workers; and hookworm, schistosomiasis, and malaria produce a “perfect storm of anemia” that reduces productive capacity.^13^ Thus, malaria and the NTDs have a pivotal role in the world's food crisis.^26^

### War.

Less obvious, but a concept that I believe to be equally important is the effects of tropical infections on promoting war and conflict.^25^,^26^ There is no question that tropical infections can arise from the breakdowns in public health infrastructure that result from war. During the 20th century, American troops returning from the Pacific Theatre of World War II had high rates of tropical diseases including more than 500,000 cases of malaria; 100,000 cases of dengue; and 10,000 cases each of lymphatic filariasis and hookworm infection.^27^

**Figure 2.**
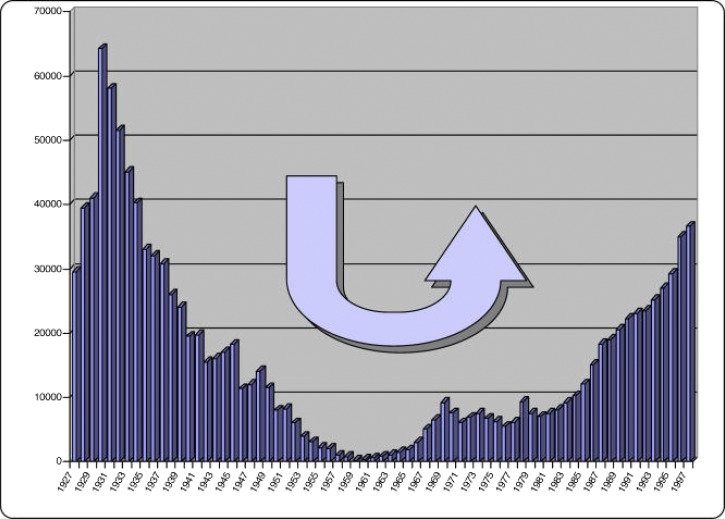
New cases of sleeping sickness reported for Africa during 1927–1997. From Simarro and others.^28^.

Civilian non-combatants also have high rates of tropical infections in conflict situations. One recent example is the high rates of human African trypanosomiasis (HAT) (sleeping sickness) as a result of years of civil and international conflict in Angola, Democratic Republic of Congo, Sudan, and elsewhere in sub-Saharan Africa beginning in the 1970s and extending until the end of the 20th century (Figure 2).^25^,^28^ This disease re-emerged when mobile health teams that previously identified and treated cases of the infection could no longer enter war-torn areas of sub-Saharan Africa and tsetse control measures were interrupted.^25^,^28^ Now that hostilities in some of these areas are reduced and control re-instated, it becomes plausible to envision the eventual elimination of HAT in Africa.^28^

Based on studies demonstrating an overlap between conflict and tropical infections, I became interested in asking whether the equation might flow the other way, in other words whether the community destabilizing effects of the NTDs outlined in Table 3 might also actually promote conflict just as they promote poverty.^26^ Understanding the relationship between “conflict and contagion” may be an important concept given the high rates of NTDs, which I found previously in “hotspot” geopolitical areas such as in the nations of the Organization of the Islamic Conference (the world's Islamic countries)^29^ and even in some large middle-income countries with nuclear weapons capabilities such as China, India, and Iran.^30^ Thus, as the U.S. military extends its activities into sub-Saharan Africa through the new U.S. African Command and as the U.S. Department of State extends its outreach in LMICs,^31^ the role of NTD control and elimination may become an important theme in U.S. foreign policy.^26^ We will return to this concept later when I discuss the prospects for science and vaccine diplomacy.

## A Call To Eliminate The Tropical Diseases

Over the past two decades enormous strides have been made in NTD control and even elimination through mass drug administration (MDA).^4^,^14^,^18^,^25^,^32^ The concepts and implementation of MDA have been pioneered by several important members of ASTMH, including many persons here with us this evening! During the 1980s and 1990s, China became one of the first large countries to eliminate lymphatic filariasis as a public health problem through MDA with the drug diethylcarbamazine citrate.^4^,^25^,^32^ The success of this enterprise in China in turn built on the pioneering work conducted by Frank Hawking (father of the cosmologist Stephen J. Hawking) and others during the 1950s and 1960s.^25^,^32^ Through widespread MDA requiring more than a billion treatments annually with low-cost diethylcarbamazine or ivermectin (Mectizan^®^) and albendazole donated by Merck & Co. and GlaxoSmithKline, respectively, lymphatic filariasis has now been eliminated as a public health problem in more than 20 LMICs.^4^ Similarly donated ivermectin has resulted in the elimination of onchocerciasis from Mali and Senegal, and will soon lead to onchocerciasis elimination in Latin America; donated azithromycin (Zithromax^®^), together with simple surgeries, facial cleansing, and environmental control, have led to trachoma elimination in more than a dozen countries.^4^ Thus, MDA has become a powerful global tool in eliminating these three devastating NTDs.

To build on the successes for lymphatic filariasis, onchocerciasis, and trachoma elimination through MDA, beginning in 2005 several of us here this evening, together with the WHO and others, began to advocate for bundling MDA for these diseases with MDA for the soil-transmitted helminthiases and schistosomiasis.^9^,^18^,^32^ Today, through such advocacy efforts along with safety data to support the simultaneous administration of albendazole, ivermectin, and praziquantel,^32^ so-called “rapid impact” packages of donated and/or low-cost drugs are being provided through national programs of MDA for the seven of the most common NTDs, namely ascariasis, trichuriasis, hookworm, schistosomiasis, lymphatic filariasis, onchocerciasis, and trachoma.^4^,^33^ Integrated NTD control and elimination efforts are now underway in almost 20 countries through the financial support of the United States Agency for International Development and their Neglected Tropical Diseases Program,^34^ the British Department for International Development,^35^ and the World Bank, as well as privately through a new END (“End Neglected Diseases”) Fund, jointly administered by Geneva Global and the Global Network for NTDs of the Sabin Vaccine Institute.^33^,^36^,^37^ The Global Network for NTDs is supported by the Gates Foundation. The ultimate goal for integrating MDA is to advance the elimination of lymphatic filariasis, trachoma, and onchocerciasis (as well as leprosy elimination through multi-drug therapy), and simultaneously reducing the prevalence of the soil-transmitted helminthiases and schistosomiasis.^4^,^32^,^33^

**Figure 3.**
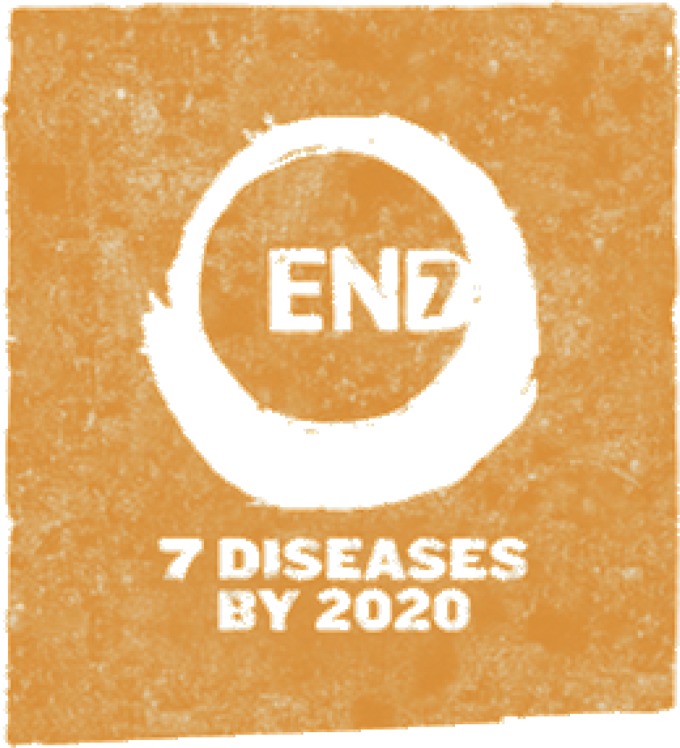
Logo of the END 7 campaign from the Global Network for Neglected Tropical Diseases of the Sabin Vaccine Institute.^34^

Towards that purpose, under the direction of Dr. Neeraj Mistry, a new “END 7” advocacy campaign has just been launched (Figure 3).^37^ The success of these NTD initiatives depends absolutely on an incredibly committed group of health ministries in the disease-endemic countries working jointly within the framework of a global NTD alliance of key technical partnerships that work with WHO and its regional offices.^33^ Such partnerships include the Research Triangle Institute International, Family Health International, the Schistosomiasis Control Initiative, Helen Keller International, the Carter Center, Deworm the World, Sight Savers, the Christian Blind Mission, the Global Alliance to Eliminate Lymphatic Filariasis, the Center for Neglected Tropical Diseases of the Liverpool School of Tropical Medicine, the African Program for Onchocerciasis Control, the Onchocerciasis Elimination Program for the Americas, the International Trachoma Initiative, Task Force for Global Health, and other organizations, and literally billions of dollars worth of pharmaceutical company donations from Merck & Co., GlaxoSmithKline, Johnson & Johnson, Pfizer Inc., Novartis, Merck KgaA, Sanofi-Aventis, and MedPharm.^33^

At the current rate of international giving and NTD control and elimination activities, we are still far from eliminating lymphatic filariasis, trachoma, and onchocerciasis through MDA, and even further from even considering the possibility of eliminating the highest prevalence NTDs such as the soil-transmitted helminthiases and schistosomiasis.^4^,^33^ An important step would be to persuade the U.S. and British Governments to expand their current donations, and possibly persuade other countries in Europe as well to begin donating.^38^ In a 2010 paper, I used a term coined by Fareed Zakaria, “The Post-American World,” to emphasize how the major emerging economies including Brazil, India, China, and even Nigeria are catching up to the U.S. and Europe and have (or will soon have) sufficient resources to begin contributing to global MDA efforts for NTDs, and simultaneously the great sovereign wealth of the Middle Eastern countries has a similar obligation.^38^ We cannot continue to rely entirely on the United States and the United Kingdom for all of the scale-up required to effect NTD control and elimination through MDA. With the economic downturn disproportionately affecting the United states and Europe, we will depend more than ever on the emerging economies and the Middle East to step up and contribute to NTD efforts.^38^ In this sense, the long-standing commitment of the government of Kuwait to onchocerciasis control and elimination is especially welcomed.

With the exceptions of lymphatic filariasis and trachoma,and possibly onchocerciasis and leprosy in some countries, we will not achieve elimination of the high prevalence NTDs through MDA even if other countries step up to contribute financial and technical support. How then should we move to accelerate NTD control and elimination efforts globally? Together with Dr. Bernard Pecoul of the Drugs for Neglected Diseases Initiative, I have argued that it is critical to move away from a concept put forward by some global health experts of the “tool-ready” NTDs, i.e., diseases currently being targeted by MDA, versus “tool-deficient” NTDs, i.e., diseases not yet amenable to MDA.^39^ Instead, we countered that all NTDs are “tool-ready” in the sense that we can go a long way towards controlling or even in some cases eliminating complicated kinetoplastid infections such as HAT, Chagas disease, and kala-azar through case detection and treatment together with integrated vector management.^39^ In parallel, almost all NTDs are “tool-deficient;” e.g., in areas of high transmission of hookworm infection and schistosomiasis, there are rapid rates of post-treatment re-infection and in many instances, such as single-dose mebendazole treatment of hookworm, MDA is simply not effective even in reducing prevalence.^39^,^40^ Therefore as a “manifesto” for accelerating a global assault on the NTDs, it is necessary to scale-up MDA in parallel with conducting research and development (R&D) to produce a new generation of appropriate technologies, including new drugs, diagnostics, and vaccines.^39^,^41^ A key point here is that we must expand MDA and international R&D efforts simultaneously.

To emphasize the importance of an agenda that embraces expansions in MDA and R&D, I propose that we look to an “audacious goal” proposed by the Gates Foundation and a community of international health agencies, scientists, and health advocates in 2007 to advance the eradication of malaria.^4^ Whether such efforts will actually lead to eradication in the sense that smallpox has been eradicated is for me less important than a key tenet of the audacious goal, which is to scale-up the use of existing anti-malaria interventions such as long-lasting insecticide treated nets, intermittent preventive therapy, and treatment with artemisinin-combination therapies, and in parallel calling on the scientific community and the industrial sector to expand R&D efforts to accelerate the development of new malaria drugs, diagnostics, vaccines, insecticides, and other appropriate technologies. For me, this goal is a brilliant concept for its audaciousness and simplicity.

I recently called on the global health community to launch a similar audacious goal for the 17 major NTDs.^4^ For example, our human hookworm vaccine under development by the Sabin Vaccine Institute is a necessary and appropriate technology given the high rates of mebendazole failure and post-treatment re-infection after albendazole chemotherapy.^40^ We are working with Dr. Simon Brooker (London School of Hygiene and Tropical Medicine) and Dr. Bruce Lee's group (University of Pittsburgh) to model on the impact of the human hookworm vaccine to interrupt and ultimately possibly effect hookworm elimination. The human hookworm vaccine targets the blood feeding stages of *Necator americanus*, the major hookworm worldwide, and is entering clinical development through support of the Gates Foundation and the Dutch Ministry of Foreign Affairs.^40^ Dr. Maria Elena Bottazzi heads our product development efforts, together with Drs. Bin Zhan, Kathryn Jones, Coreen Beaumier, Elena Curti, and Chris Seid, as well as Portia Gillespie, Brian Keegan, Cliff Kwityn, and Wanderson Rezende. In parallel, the clinical development is being led by Dr. David Diemert with the assistance of Dr. Shannon Grahek and an extensive staff in Minas Gerais State, Brazil; quality assurance is headed by Marva Loblack, Angela Oliver, and Cheryl Basile; program management by Carla Crooks; and the clinical immunology laboratory is headed by Dr. Jeff Bethony, together with Dr. Amar Jariwala. Drs. Alex Loukas and Mark Pearson are key partners at James Cook University in Australia. Dr. Rodrigo Correa-Oliveira oversees our hookworm vaccine operations in Brazil in his role as director of Fiocruz in Belo Horizonte.

To manufacture the two antigens of the human hookworm vaccine, we work with industrial partners at Aeras (Rockville, MD) under the direction of Jim Connolly; Fraunhofer Center for Molecular Biology (Newark, DE), headed by Dr. Vidadi Yusibov; FIOCRUZ–Center for Technological Development in Health (headed by Drs. Carlos Morel); FIOCRUZ BioManguinhos (Brazil), headed by Drs. Akira Homma, Artur Roberto Coutu, and Marcos Friere; as well as an Executive Board comprised of Drs. L. Russell and C. de Quadros, and Mike Whitham. Through support of Len Blavatnik, Mort Hyman, and the National Institute of Allergy and Infectious Diseases/National Institutes of Health, the Sabin Vaccine Institute is also working to develop new vaccines for intestinal schistosomiasis together with some of the same partners listed above, in addition to Brazil's Instituto Butantan (Drs. Jorge Kalil, Isaias Raw and Beth Martin).^40^ Through support of the Carlos Slim Health Institute (Drs. Roberto Tapia-Conyer and Miguel Betancourt Cravioto) a therapeutic Chagas disease vaccine based on discoveries made in the laboratory of Dr. Eric Dumonteil at the Autonomous University of Yucatan is at an earlier stage of development.^42^

In collaboration with Dr. Michael Heffernan of BCM, we are exploring how nanoparticle technology may one day enhance the immunological potency of a Chagas vaccine to stimulate effector CD8+ T cells.^42^ A leishmaniasis vaccine is also under development in collaboration with the National Institute of Allergy and Infectious Diseases/National Institutes of Health laboratory of Drs. Jesus Valenzuela and Shaden Kamhawi, and we have embarked on new collaborations for vaccines to combat severe acute respiratory syndrome with Drs. Sara Lustigman and Shibo Jiang (New York Blood Center) and Kent Chien-Te (University of Texas Medical Branch), and now a new biodefense vaccine portfolio with Dr. Brett Giroir (National Center for Biosecurity at Texas A&M University).

Sabin's NTD vaccines, also known as ‘antipoverty' vaccines, are part of a larger global portfolio established by a group of PDPs and other pharmaceutical companies (Table 4 ).^1^ PDPs are non-profit organizations that use industry practices to advance product development. Globally, more than 20 different PDPs are involved in drug, diagnostic, vaccine, microbiocide, and insecticide development for tropical diseases in addition to new products for HIV/AIDS, tuberculosis, lower respiratory-tract infections, and diarrhea. The Seattle-based Infectious Diseases Research Institute and the Korea-based International Vaccine Institute are also producing NTD vaccines, and the Program for Appropriate Technology in Health Vaccines is a PDP in Washington, DC, that has pioneered the licensure of a new vaccine for meningococcus A and phase 3 studies for a malaria vaccine jointly with GlaxoSmithKline.

The elimination of the 17 major NTDs will require expanded MDA together with the development, manufacture, testing, delivery, and judicious use of the new antipoverty drugs and vaccines anticipated to come on-line over the next decade. The scientific hurdles to develop such life-saving products aside, we face the looming problem of how to fund R&D for new products and their industrial scale manufacture. Recently, I advocated for allocating 1–2% of President Obama's Global Health Initiative, which would infuse US$100–200 million into the system to support antipoverty products.^41^ The Global Health Initiative support of NTD products would require co-support through global R&D funds provided by emerging economies and the Middle East. Critical allies in the development of new antipoverty vaccines will be collaborations between the PDPs and a group of vaccine manufacturers belonging to the Developing Countries Vaccine Manufacturers Network (DCVMN).^42^ For development of hookworm and schistosomiasis vaccines, the Sabin Vaccine Institute collaborates with DCVMN members, FIOCRUZ BioManguinhos and Instituto Butantan; and we work with the Centro de Investigación y de Estudios Avanzados del Instituto Politécnico Nacional in Mexico (Dr. Jaimie Ortega) and Birmex (Dr. Samuel Ponce de Leon Rosales) on the Chagas disease vaccine. Such partnerships will be critical for ensuring global access for antipoverty vaccines. The DCVMN represents a potent force for ensuring manufacture of new antipoverty vaccines for the bottom billion. They will be essential for realizing the vision of this “Decade of Vaccines” that was announced in 2010 by the Gates Foundation.^1^

## Science Diplomacy And The Global Fight

Manufacturing vaccines in partnership with developing country vaccine manufacturers may also have a profound geopolitical dimension. To understand this concept, we must look to the legacy of Dr. Albert Sabin, who during the late 1950s developed the oral polio vaccine jointly with Soviet virologists. This period coincided with an apex in the Cold War in the years immediately after the launch of Sputnik and the first successful test of a hydrogen bomb by the Soviet Union. Despite heightened political tensions, the polio collaboration provided proof-of-concept for how two nations can set aside ideologies for purposes of vaccine development, something that I have termed “vaccine diplomacy.”^43^,^44^ Based on our successful collaborations with members of the DCVMN, I have started to ask if there might be modern day examples of vaccine diplomacy, particularly with nations that 1) often differ ideologically with the United States, 2) have capacity for vaccine development and production, and 3) simultaneously have high rates of NTDs. Among the more poignant examples would be NTD-endemic Islamic nations of strategic security interests to the United States, such as Indonesia, Iran, and Pakistan.^43^,^44^ A number of countries where vaccine diplomacy might one day be practiced is shown in Table 5 . Such collaborations will be fraught with political challenges but potentially they also offer huge rewards.

Collaborations with the DCVMN are not merely the United States helping developing countries produce vaccines for LMICs. The United States also has extensive poverty with some estimates suggesting that up to 50 million Americans now live below the poverty line, especially in areas such as Texas and the U.S. Gulf Coast. Our studies have shown that surprising rates of some NTDs travel with poverty in these regions.^3^,^45^,^46^ Accordingly, Drs. Laila Woc-Colburn, Jose Serpa, and Bob Parkerson at BCM have established the tropical medicine clinic at the public hospital linked with BCM, and are now seeing patients with variety of NTDs.^47^ Our National School of Tropical Medicine has key elements for tropical medicine R&D, clinical practice, and training, similar to a concept I advanced in 2008 when President Obama indicated his desire to close the detainee facility at Guantanamo.^48^

Worldwide, wherever there is poverty we can identify high rates of NTDs. In my opinion, of all external factors influencing the rates of tropical diseases, poverty trumps all. Tropical infections thrive in the setting of poverty and they promote poverty. However, it does not have to be this way. Although global poverty will require decades or even centuries to eradicate, globally we can break this vicious cycle as we now have the technical ability to eliminate all of the high prevalence NTDs as public health problems. It will require an unprecedented level of international cooperation to expand current levels of MDA coverage and scientific collaborations to produce new drugs, diagnostics, vaccines, and insecticides as necessary.^4^ I believe an important role for our ASTMH will be to continue serving as a neutral forum for scientific debate and discussion on the global elimination of tropical diseases. It has been an honor to serve as your President this past year. Thank you.

## Figures and Tables

**Table 1:** Plague and pestilence: Number of global cases of tropical diseases*

Disease	Estimated no. cases
Ascariasis	807 million
Trichuriasis	604 million
Hookworm infection	576 million
Schistosomiasis	391–587 million
Amebiasis†	480 million
Malaria	216 million
Lymphatic filariasis	115 million
Dengue	70–500 million
Trachoma	40 million
Strongylodiasis	30–100 million
Onchocerciasis	26 million
Liver fluke infection‡	24 million
Paragonimiasis	23 million
Typhoid fever	22 million
Leishmaniasis	12 million
Chagas disease	10 million
Intestinal fluke infection	7 million
Paratyphoid fever	5 million
Cholera	3–5 million
Fascioliasis	3 million
Leprosy	< 0.5 million
Total	3.5–4.2 billion

* Based on references 4–16.

† It is likely that a large but unknown percentage of these cases are from non-pathogenic *Entamoeba dispar* rather than invasive *E. histolytica* infections.

‡ Combined clonorchiasis and opisthorchiasis.^5^

**Table 2:** Death: Annual number of global deaths from tropical diseases*

Disease	Estimate no. deaths
Malaria	655,000
Schistosomiasis	280,000†
Typhoid fever	217,000
Cholera	120,000
Hookworm infection	65,000
Rabies	55,000
Leishmaniasis	51,000
Amebiasis	40,000
Dengue	21,000
Chagas disease	14,000
Trichuriasis	10,000
Food-borne trematodiases	7,000
Leprosy	6,000
Total	1.5 million

* Based on references Refs 5–8, 15–17, and 21–23.

† Sub-Saharan Africa only.

**Table 3:** Famine and war: Food insecurity and conflict-exacerbating elements of tropical diseases*

Reductions in agricultural productivity
Abandonment of agricultural lands
Pivotal role in world's food crisis
Reductions in education and future wage-earning
Promotion of ignorance and stigma
Adverse child and maternal health
Adverse child and maternal health

* Modified from Hotez and Thompson.^26^

**Table 4:** Vaccines needed for neglected tropical diseases over the next decade*

Disease	Type	Manufacturer or status
African trypanosomiasis	Veterinary	Fraunhofer Center for Molecular Biotechnology
Buruli ulcer	Human preventive or therapeutic	Not currently available
Chagas disease	Human preventive or therapeutic	Sabin Vaccine Institute
Cysticercosis	Veterinary	Indian Immunologicals (Indimmune)
Dengue	Human preventive	GlaxoSmithKline, Merck & Co., Sanofi Pasteur
Echinococcosis	Veterinary	Not currently available (academic institutions only)
Hookworm infection	Human preventive	Sabin Vaccine Institute
Leishmaniasis	Human preventive or therapeutic	Infectious Disease Research Institute
Leprosy	Human therapeutic	Infectious Disease Research Institute
Liver fluke	Human preventive	Not currently available
Onchocerciasis	Human preventive	Sabin Vaccine Institute
Rabies	Human post-exposure	Novartis, Sanofi Pasteur
Schistosomiasis	Human preventive	Institute Pasteur, Sabin Vaccine Institute

* Modified from Hotez.^1^

**Table 5:** Opportunities for vaccine diplomacy for the United States

Region	Country
Middle East	Iran, Saudi Arabia
Eastern Asia	China, India, Indonesia, Malaysia, North Korea, Pakistan, Singapore, Thailand, Vietnam
Latin America	Brazil, Cuba, Mexico
Africa	Senegal, South Africa

## References

[1] Hotez P (2011). A handful of ‘antipoverty' vaccines exist for neglected diseases, but the world's poorest billion people need more. Health Aff (Millwood).

[2] Hotez P (2010). A national school of tropical medicine and neglected infections of poverty for North America. PLoS Negl Trop Dis.

[3] Hotez PJ, Bottazzi ME, Dumonteil E, Kamhawi E, Valenzuela J, Ortega J, Ponce de Leon Rosales S, Betancourt Cravioto M, Tapia-Conyer R (2012). Texas and Mexico: sharing a legacy of poverty and neglected tropical diseases. PLoS Negl Trop Dis.

[4] Hotez P (2011). Enlarging the “audacious goal:” elimination of the world's high prevalence neglected tropical diseases. Vaccine.

[5] Furst T, Keiser J, Utzinger J (2012). Global burden of human food-borne trematodiasis: a systematic review and meta-analysis. Lancet Infect Dis.

[6] Thomas SJ, Endy TP (2011). Vaccines for the prevention of dengue: development update. Hum Vaccin.

[7] *World Health Organization Fact Sheets* http://www.who.int/mediacentre/factsheets/fs107/en/.

[8] World Health Organization (2011). World Malaria Report 2011.

[9] World Health Organization (2010). Working to Overcome the Global Impact of Neglected Tropical Diseases: the First WHO Report on Neglected Tropical Diseases.

[10] King CH (2010). Parasites and poverty: the case of schistosomiasis. Acta Trop.

[11] Olsen A, van Lieshout L, Marti H, Polderman T, Polman K, Steinmann P, Stothard R, Thybo S, Verweij JJ, Magnussen P (2009). Strongyloidiasis: the most neglected of the neglected tropical diseases. Trans R Soc Trop Med Hyg.

[12] Burton MJ, Mabey DW (2009). The global burden of trachoma: a review. PLoS Negl Trop Dis.

[13] Hotez PJ, Fenwick A, Savioli L, Molyneux DH (2009). Rescuing the bottom billion through control of neglected tropical diseases. Lancet.

[14] Hotez PJ, Molyneux DH, Fenwick A, Kumaresan J, Ehrlich Sachs S, Sachs JD, Savioli L (2007). Control of neglected tropical diseases. N Engl J Med.

[15] Crump JA, Luby SP, Mintz ED (2004). The global burden of typhoid fever. Bull World Health Organ.

[16] Walsh JA (1986). Problems in recognition and diagnosis of amebiasis: estimation of the global magnitude of morbidity and mortality. Rev Infect Dis.

[17] Hotez PJ, Molyneux DH, Fenwick A, Ottesen E, Ehrlich Sachs S, Sachs JD (2006). Incorporating a rapid-impact package for neglected tropical diseases with programs for HIV/AIDS, tuberculosis, and malaria. PLoS Med.

[18] Molyneux DH, Hotez PJ, Fenwick A (2005). “Rapid-impact interventions:” how a policy of integrated control for Africa's neglected tropical diseases could benefit the poor. PLoS Med.

[19] Hotez PJ, Ottesen E, Fenwick A, Molyneux D (2006). The neglected tropical diseases: the ancient afflictions of stigma and poverty and the prospects for their control and elimination. Adv Exp Med Biol.

[20] Hotez PJ (2009). Empowering women and improving female reproductive health through control of neglected tropical diseases. PLoS Negl Trop Dis.

[21] World Health Organization (2002). WHO Expert Committee. Prevention and control of schistosomiasis and soil-transmitted helminthiasis. World Health Organ Tech Rep Ser.

[22] Van der Werf MJ, de Vlas SJ, Brooker S, Looman CW, Nagelkerke NJ, Habbema JD, Engels D (2003). Quantification of clinical morbidity associated with schistosome infection in sub-Saharan Africa. Acta Trop.

[23] Black RE, Cousens S, Johnson H, Lawn JE, Rudan I, Bassani DG, Jha P, Campbell H, Fischer Walker C, Cibulskis R, Eisele T, Liu L, Mathers C, for the Child Health Epidemiology Reference Group of WHO and UNICEF (2010). Global, regional, and national causes of child mortality in 2008: a systematic analysis. Lancet.

[24] *UNAIDS* http://www.unaids.org/en/dataanalysis/.

[25] Hotez PJ (2008). Forgotten People, Forgotten Diseases: The Neglected Tropical Diseases and their Impact on Global Health and Development.

[26] Hotez PJ, Thompson TG (2009). Waging peace through neglected tropical disease control: a US foreign policy for the bottom billion. PLoS Negl Trop Dis.

[27] Mackie TT (1947). Tropical disease problems among veterans of World War II: preliminary report. Trans Am Clin Climatol Assoc.

[28] Simarro PP, Jannin J, Cattand P (2008). Eliminating human African trypanosomiasis: where do we stand and what comes next. PLoS Med.

[29] Hotez PJ (2009). The neglected tropical diseases and their devastating health and economic impact on the member nations of the Organisation of the Islamic Conference. PLoS Negl Trop Dis.

[30] Hotez PJ (2010). Nuclear weapons and neglected diseases: the “ten thousand-to-one gap.”. PLoS Negl Trop Dis.

[31] Hotez PJ (2011). Unleashing “civilian power”: a new American diplomacy through neglected tropical disease control, elimination, research, and development. PLoS Negl Trop Dis.

[32] Hotez PJ (2009). Mass drug administration and the integrated control of the world's high prevalence neglected tropical diseases. Clin Pharmacol Ther.

[33] Hotez PJ (2010). A plan to defeat neglected tropical diseases. Sci Am.

[34] *USAID's Neglected Tropical Disease Program* http://www.neglecteddiseases.gov/.

[35] *Neglected Tropical Diseases* http://www.dfid.gov.uk/What-we-do/Key-Issues/Health/Neglected-tropical-diseases/.

[36] *Geneva Global Inc., African Health Fund* http://www.genevaglobal.com/funds-african-health.php.

[37] *Global Network for Neglected Tropical Diseases* http://www.globalnetwork.org/.

[38] Hotez PJ (2010). Neglected tropical disease control in the “Post-American World.”. PLoS Negl Trop Dis.

[39] Hotez PJ, Pecoul B (2010). “Manifesto” for advancing the control and elimination of neglected tropical diseases. PLoS Negl Trop Dis.

[40] Hotez PJ, Bethony JM, Diemert DJ, Pearson M, Loukas A (2010). Developing vaccines to combat hookworm infection and intestinal schistosomiasis. Nat Rev Microbiol.

[41] Hotez PJ (2011). New antipoverty drugs, vaccines, and diagnostics: a research agenda for the US President's Global Health Initiative (GHI). PLoS Negl Trop Dis.

[42] *DVCMN* http://www.dcvmn.org/index.aspx.

[43] Hotez PJ (2010). Peace through vaccine diplomacy. Science.

[44] Hotez P (2011). Engaging Iran through vaccine diplomacy.

[45] Hotez PJ (2008). Neglected infections of poverty in the United States of America. PLoS Negl Trop Dis.

[46] Hotez PJ (2011). America's most distressed areas and their neglected infections: the United States gulf coast and the District of Columbia. PLoS Negl Trop Dis.

[47] Hotez P (2011). A new tropical medicine clinic for ‘third world America.'.

[48] Hotez PJ (2008). Reinventing Guantanamo: from detainee facility to center for research on neglected diseases of poverty in the Americas. PLoS Negl Trop Dis.

